# Galectin-3, histone deacetylases, and Hedgehog signaling: Possible convergent targets in schistosomiasis-induced liver fibrosis

**DOI:** 10.1371/journal.pntd.0005137

**Published:** 2017-02-23

**Authors:** Felipe Leite de Oliveira, Katia Carneiro, José Marques Brito, Mariana Cabanel, Jonathas Xavier Pereira, Ligia de Almeida Paiva, Wingkin Syn, Neil C. Henderson, Marcia Cury El-Cheikh

**Affiliations:** 1 Instituto de Ciências Biomédicas, Universidade Federal do Rio de Janeiro, Rio de Janeiro, Brazil; 2 Departmento de Fisiologia e Farmacodinâmica, Instituto Oswaldo Cruz, Rio de Janeiro, Brazil; 3 Section of Gastroenterology, Ralph H Johnson Veterans Affairs Medical Center, Charleston, South Carolina, United States of America; 4 Division of Gastroenterology and Hepatology, Medical University of South Carolina, Charleston, South Carolina, United States of America; 5 MRC Centre for Inflammation Research, The Queen’s Medical Research Institute, University of Edinburgh, Edinburgh, United Kingdom; Universidade Federal de Minas Gerais, BRAZIL

## Abstract

Schistosomiasis affects approximately 240 million people in the world. *Schistosoma mansoni* eggs in the liver induce periportal fibrosis and hepatic failure driven by monocyte recruitment and macrophage activation, resulting in robust Th2 response. Here, we suggested a possible involvement of Galectin-3 (Gal-3), histone deacetylases (HDACs), and Hedgehog (Hh) signaling with macrophage activation during Th1/Th2 immune responses, fibrogranuloma reaction, and tissue repair during schistosomiasis. Gal-3 is highly expressed by liver macrophages (Kupffer cells) around *Schistosoma* eggs. HDACs and Hh regulate macrophage polarization and hepatic stellate cell activation during schistosomiasis-associated fibrogenesis. Previously, we demonstrated an abnormal extracellular matrix distribution in the liver that correlated with atypical monocyte–macrophage differentiation in *S*. *mansoni*-infected, Gal-3-deficient (Lgals3-/-) mice. New findings explored in this review focus on the chronic phase, when wild-type (Lgals3+/+) and Lgals3-/- mice were analyzed 90 days after *cercariae* infection. In Lgals3-/- infected mice, there was significant inflammatory infiltration with myeloid cells associated with egg destruction (hematoxylin and eosin staining), phagocytes (specifically Kupffer cells), numerically reduced and diffuse matrix extracellular deposition in fibrotic areas (Gomori trichrome staining), and severe disorganization of collagen fibers surrounding the *S*. *mansoni* eggs (reticulin staining). Granuloma-derived stromal cells (GR cells) of Lgals3-/- infected mice expressed lower levels of alpha smooth muscle actin (α-SMA) and eotaxin and higher levels of IL-4 than Lgals3+/+ mice (real-time PCR). The relevant participation of macrophages in these events led us to suggest distinct mechanisms of activation that culminate in defective fibrosis in the liver of Lgals3-/- infected mice. These aspects were discussed in this review, as well as the possible interference between Gal-3, HDACs, and Hh signaling during progressive liver fibrosis in *S*. *mansoni*-infected mice. Further studies focused on macrophage roles could elucidate these questions and clear the potential utility of these molecules as antifibrotic targets.

## Schistosomiasis and the establishment of liver fibrosis

Parasitic infections are one of the most common causes of liver diseases in humans [1]. Helminthes of the genus Schistosoma cause a neglected tropical disease that affects approximately 240 million people, mainly in developing countries. The three major schistosomes infecting humans are *S*. *mansoni*, *S*. *japonicum*, and *S*. *haematobium*. *S*. *mansoni* is most commonly found in the endemic areas of Africa and Latin America and is a major public health concern because it often infects people who are likely to swim in fresh water containing snails that harbor sporocysts and release *cercariae* (the larval infective form of the parasite [2]) in the water. *S*. *mansoni* infection leads to periportal fibrosis, portal hypertension, ascites, and gastrointestinal bleeding correlated with bloody diarrhea, abdominal pain, right upper quadrant pain, and esophageal varices, resulting in severe symptoms and death in approximately 60% of infected patients [3,4]. Although the World Health Organization introduced regulations to control the growth of schistosomiasis, its prevalence is significantly high in some countries, including Brazil and China [5,6].

Schistosomiasis mansoni leads to liver diseases because of egg deposition in the periportal zones, which induces a granulomatous reaction that culminates in liver fibrosis and portal hypertension [7]. The prehepatic phase is preceded by cercarial penetration through the skin with significant changes in the epidermis [8]. After skin penetration, the infecting form transforms into schistosomules that reach the lungs and later accumulate in the hepatic portal system as adult worms, where they release large numbers of eggs [9]. Some of these eggs cause abscesses in the intestinal wall, some are expelled into intestinal lumen, and the remaining eggs are washed through the portal blood flow into the liver. After reaching the intrahepatic portal system, they bring about a switch from an exudative to a progressive fibrogranulomatous inflammatory reaction [10,11,12].

At the beginning of the eggs’ deposition, their antigens induce a local production of inflammatory cytokines (IL-1 and TNF-α) from resident epithelial cells and macrophages that trigger the early influx of monocytes and neutrophils, forming a macrophage-dependent granulomatous inflammatory reaction [13,14,15,16]. At the systemic level, there is a moderate Th1 response against schistosomula-maturing worms antigens that is crucial to mobilize blood monocytes from bone marrow towards the eggs trapped into the mesenteric venous system [10]. It has been shown in the literature that blood monocytes are a heterogeneous population based primarily on the amount of time they spend in the blood before migrating into tissues [17,18]. For instance, it has been described that proinflammatory (Ly6C^low^CD11b^+^CCR2^+^CX3CR1^−^F4/80^+^CD62L^−^CD43) and profibrotic monocyte (Ly6C^high^CD11b^+^CCR2^−^CX3CR1^+^F4/80^+^CD43^−^) phenotypes may be modulated, regulating the initiation, maintenance, and resolution of chronic inflammatory response [17,18]. Thus, the macrophages play a critical role at the beginning of exudative granulomas formation, modulating the behavior of resident epithelial cells and quiescent hepatic stellate cells [19]. In contrast to Th1 response caused by worms antigens, the eggs antigens evoke a strong Th2 response that maintains the vigorous exudative granulomas until 8 weeks post-*cercariae* penetration [10,20,21,22], when the acute phase is downregulated and then replaced by a persistent chronic phase. During this transitory phase, the continuous egg deposition in the intrahepatic mesenteric system did not compromise the liver parenchyma. This barrier is ensured mainly by macrophages that switch from protective role at the acute phase to a wound-healing pattern, driving a concentric fibrotic reaction, which is a hallmark of the chronic phase [23]. In addition, we also noticed the following during the chronic phase: hepatosplenomegaly, polyclonal B cell activation, extra medullar hematopoiesis, and peritoneal macrophage hyperactivity [24].

## Gal-3 binds to Schistosoma and egg antigens and promotes liver fibrosis: A central role of macrophages

Gal-3 is a β-galactoside binding protein that regulates cellular activation, migration, survival, and immune response in distinct experimental models [25,26,27]. Hsu et al. (1999) showed that Gal-3 expression was absent in normal hepatocytes but was abundant in hepatocellular carcinoma, and its inhibition in these malignant cells reduced the attachment to laminin and liver metastasis [28]. Lee et al. (2002) suggested that both IL-10 and Gal-3 could cooperatively protect liver cells from ischemia reperfusion injury, controlling NO production as well as protecting the cell from injury [29]. In humans, Gal-3 is significantly accumulated in cirrhotic livers, and high levels of the serum are associated with alcoholic liver diseases [28], suggesting a direct correlation with the fibrotic stage.

In the experimental model of hepatic injury, Gal-3 has been shown to regulate the induction and resolution of hepatic fibrosis. Using Lgals3-/- mice, the data suggests that Gal-3 is required for TGF-beta-mediated myofibroblast activation and matrix production [30]. In this context, the inflammatory process involved an acute hepatocytes lesion induced by carbon tetrachloride (CCl4). In this sense, the hepatic fibrosis is installed in a short period of time compared with the long-lasting chronic inflammatory process.

Gal-3 has also been shown to induce an immune response against *S*. *mansoni*; this lectin is one of the major ligands of the polylactosamines, including GalNAc_1-4(Fuc_1–3)GlcNAc(Lac-DiNAc) structures (*N*-acetylgalactosamine _1–4 *N*-acetylglucosamine) synthesized by the worms and their eggs. As Gal-3 is highly expressed in inflammatory cells surrounding liver granulomas, it could be considered a pathogen-associated molecular pattern receptor, favoring an interactive network between macrophages and immunogenic carbohydrates [31].

In long-lasting inflammatory reactions using Lgals3-/- mice, it has been shown that macrophage share is not properly differentiated in liver granulomas and in lymphoid organs involved with the disease, such as mesenteric lymph nodes and spleen [32,33,34]. Indeed, the absence of Gal-3 also interfered with the function of Kupffer cells, the resident liver macrophages. They have a high capacity to phagocyte the secreta and excreta of the worms, becoming pigmented cells and secreting mainly IL-4 and IL-13 during the infection [11,35].

**Fig 1:**
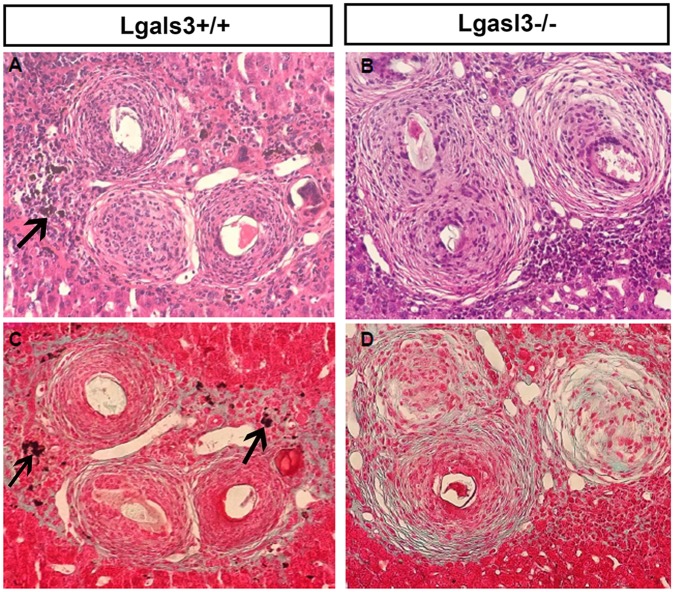
Local Kupffer cells show a decreased phagocytic activity in *S*. *mansoni*-infected Lgals3-/- mice. Classical pigmented Kupffer cells were not observed around granulomas from Lgals3-/- mice (B,D) in contrast to a high phagocytic activity found in Lgals3+/+ mice (A,C). A-B: Hematoxylin & eosin staining. C-D: Gomori trichrome staining. Magnification: 400x. *n* = 5 mice per group.

Although we have not used specific cell markers to identify these phagocytic cells, we observed that pigmented macrophages were abundant around the eggs in Lgals3+/+-infected mice, but were rarely found in livers of Lgals3-/- mice (Fig 1). Although we have not investigated if Gal-3 directly affects Kupffer cell numbers or phagocytic functions, these data demonstrate the correlation between matrix composition and Gal-3 expression. Future studies will be necessary to elucidate if and how Gal-3 modulates monocyte–macrophage functions in Schistosomiasis-associated liver fibrogenesis.

**Fig 2:**
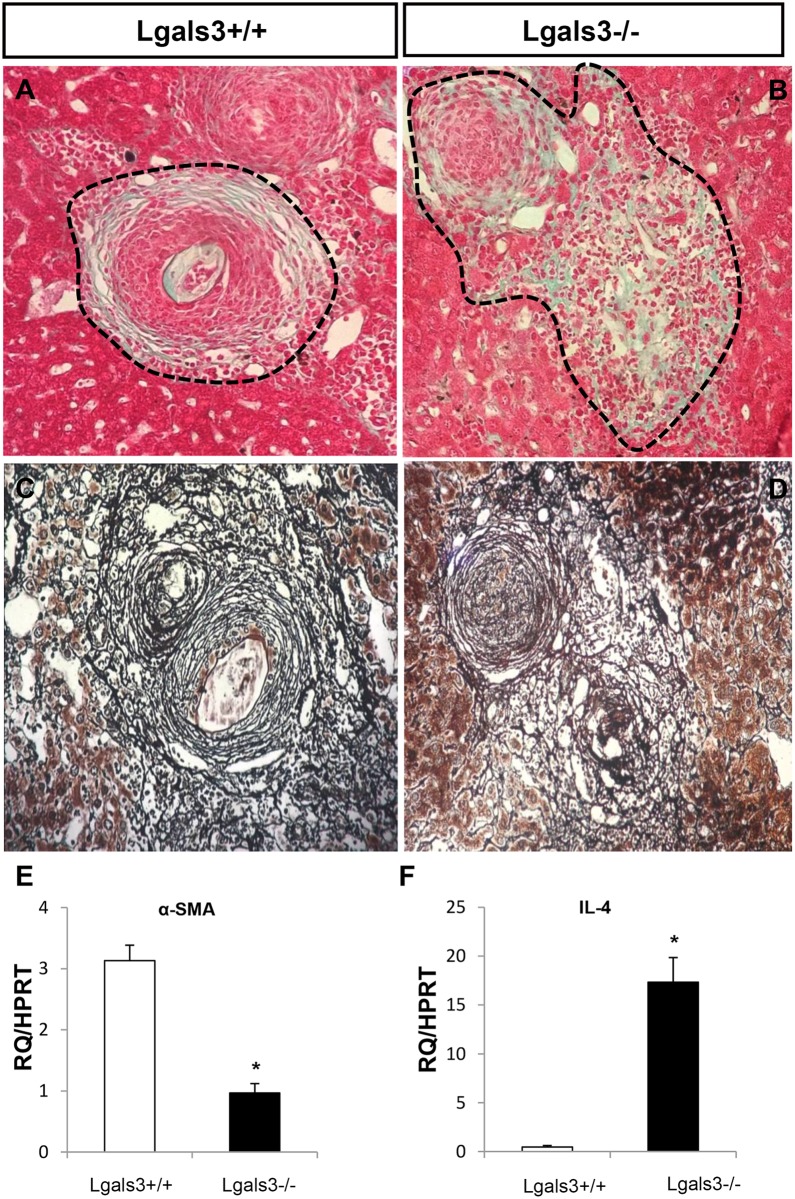
Gal-3 is necessary for fibrotic concentric granulomas in *S*. *mansoni* infected mice and myofibroblast activation. Hematoxylin & eosin and Gomori trichrome staining show classical concentric fibrotic granulomas of Lgals3+/+ (A,C dashed line) and more diffuse and dispersed collagen fibers around the *S*. *mansoni* eggs of Lgals3-/- mice (B,D dashed line). Real-time reverse transcription PCR (RT-PCR) to alpha smooth muscle actin (E) and IL-4 (F) expressed by granuloma-hepatic stellate cells (GR-HSCs) from Lgals3+/+ and Lgals3-/- mice shows the imbalance between extracellular matrix production and proinflammatory cytokines. The relative value was obtained in relation to the Hypoxanthine Guanine Phosphoribosyl Transferase (HPRT) expression. Each bar represents the mean ±SEM, *n* = 5. These graphs are representative of three experiments. *P≤0.05 compared with Lgals3+/+ mice. Magnification: 400x. *n* = 5 mice per group.

Concomitantly with this low phagocytic capacity of Kupffer cells in Lgals3-/- infected mice, there is a systemic delay in monocyte–macrophage differentiation in the periovular fibrogranulomatous reaction [32]. It drastically affects the granuloma organization, which is characterized by thinner collagen fibers abnormally diffuse throughout the liver parenchyma. Indeed, we confirmed that in the absence of Gal-3, the extracellular matrix deposition was widely dispersed throughout the liver parenchyma and loosely concentric around the egg deposition (Fig 2). These data strongly suggest that Gal-3-dependent macrophage activation could interfere with the outcome of the inflammatory process, modifying the pattern of extracellular matrix deposition by the hepatic stellate cell (HSC), maintaining, at least in part, the hepatocyte integrity. Thus, altogether, these data reinforce the role of Gal-3 in macrophage phagocytosis, described firstly by Sano et al., placing these cells as central players in both innate and adaptive immunity and modulating early and late stages of the inflammatory process [36].

## Gal-3 and hepatic stellate cells activation

In addition to the role of macrophages in granulomatous reaction, there is a relevant function for HSCs characterized by an excessive and continuous extracellular matrix deposition leading to liver injury. As a result, the HSCs change from the quiescent to an activated myofibroblastic phenotype and should be considered primary targets for the development of new antifibrotic therapies [37,38]

In fact, Gal-3 has been also shown to play a key role in liver fibrosis by mediating HSC activation and procollagen expression [30,38]. HSCs derived from chronic granulomas of *S*. *mansoni*-infected mice (GR-HSC) have been previously characterized by our group as myofibroblast cells with an important role during granulomatous reaction. As immunoregulatory cells, they produce proinflammatory cytokines that amplify the local response against the eggs antigens, contributing significantly to the pathogenesis of schistosomiasis [37,39].

In addition, we observed that GR-HSCs of Lgals3-/- mice expressed reduced levels of α-SMA compared to those isolated from granulomas of Lgals3+/+ infected mice (Fig 2E and 2F). In accordance, Jiang et al. (2012) demonstrated that HSCs from Lgals3-/- mice displayed reduced phagocytic activity and reduced expression of α-SMA and procollagen α1. The authors have shown that the extracellular Gal-3 has a key role in liver fibrosis, interfering directly with HSC function [38]. The lower levels of α-SMA expression by HSCs in the absence of Gal-3 indicate that Gal-3 positively regulates profibrotic events in the liver. On the other hand, histological analysis revealed that a diffuse patter of collagen fibers and fibrotic areas were spatially increased in these knockout mice, suggesting a more extensive region with extracellular matrix deposition in the liver of Lgals3-/- infected mice in comparison with Lgals3+/+ infected mice. In this context, we can suggest that, in the absence of macrophages activation, the GR-HSCs are less activated and, consequently, less synthetic.

Our data suggest that a high expression of IL-4 in isolated GR-HSCs from Lgals3-/- reinforces the immunomodulatory role of these cells, interfering with Th1/Th2 balance during the chronic phase of infection (Fig 2F). In accordance, IL-4αR-/- mice (nonresponsive to both IL-4 and IL-13) developed only minimal hepatic granulomas and fibrosis [40], confirming that both cytokines are critical to induce the Th2 response in schistosomiasis [14].

**Fig 3:**
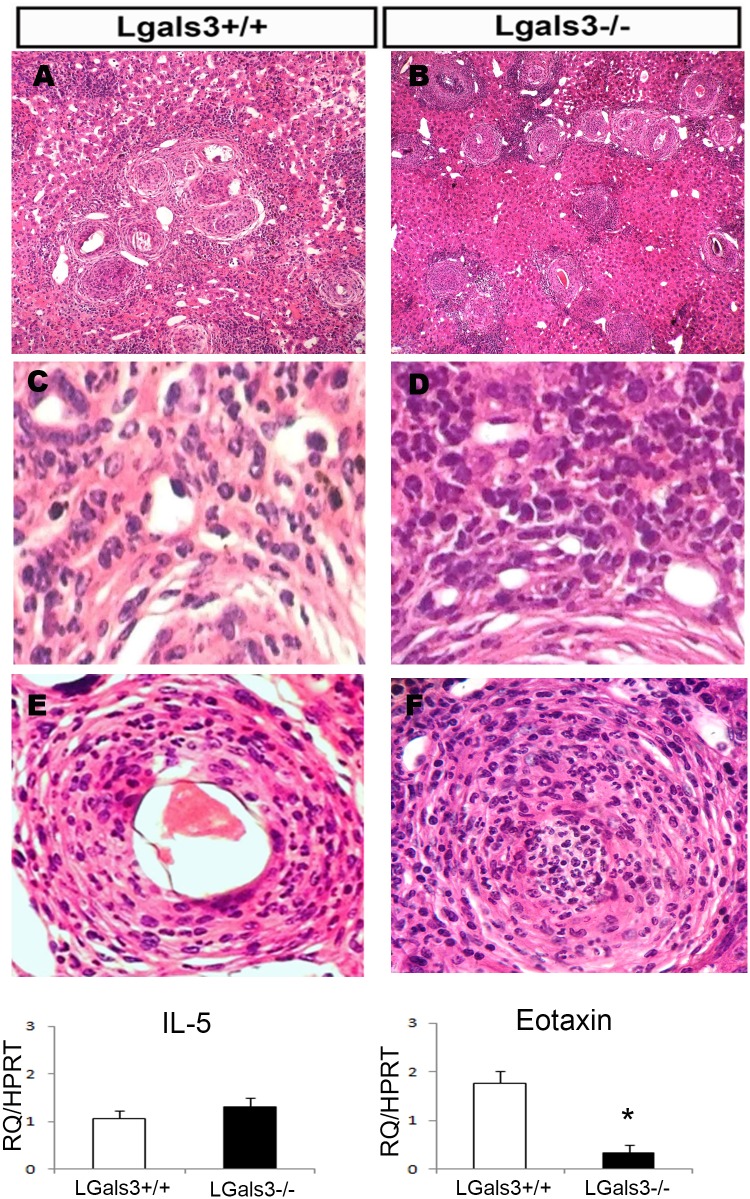
Inflammatory infiltrate is amplified in the absence of Gal-3 in *S*. *mansoni*-infected mice. Hematoxylin & eosin staining show granulomas of Lgals-3+/+ mice (A) and Lgals-3-/- mice (B), where abundant myeloid cells were detected around the eggs. Myeloid cells were preferentially localized around the eggs in Lgals-3+/+ mice (C,E). In contrast, myeloid cells were abundant around the eggs and inside the hepatic parenchyma in Lgals-3-/- mice (D,F), suggesting a local amplification instead of central mobilization. Hematoxylin & eosin staining. Real-time RT-PCR to IL-5 (G) and Eotaxin (H) expressed by GR-HSCs from Lgals-3+/+ and Lgals-3-/- mice reinforce the hypothesis of local myeloid amplification. The relative value was obtained in relation to HPRT expression. Each bar represents the mean ±SEM, *n* = 5. These graphs are representative of three experiments. *P≤0.05 compared with Lgals3+/+ mice. Magnification: A-B: 100x; C-F: 1,000x. *n* = 5 mice per group.

Another hallmark of experimental murine schistosomiasis raised by our studies is a significant inflammatory infiltrate on periovular granulomas in the chronic phase (Fig 3A and 3B). In contrast to Lgals3+/+ mice, in which mononuclear cells were predominantly observed surrounding the eggs (Fig 3C and 3E), in Lgals3-/- mice, we observed that polymorphonuclear leucocytes were the predominant cells, organized in well-defined niches or clusters (Fig 3D). This scenario suggests a peripheral amplification in response (at least in part) to local cytokines and to stromal supportive GR-HSCs. It is interesting to point out the presence of myeloid progenitor cells in contact with the hepatocytes, indicating a spreading of these cells out of the periovular granulomas in Lgals3-/- mice (Fig 3D and 3F).

**Fig 4:**
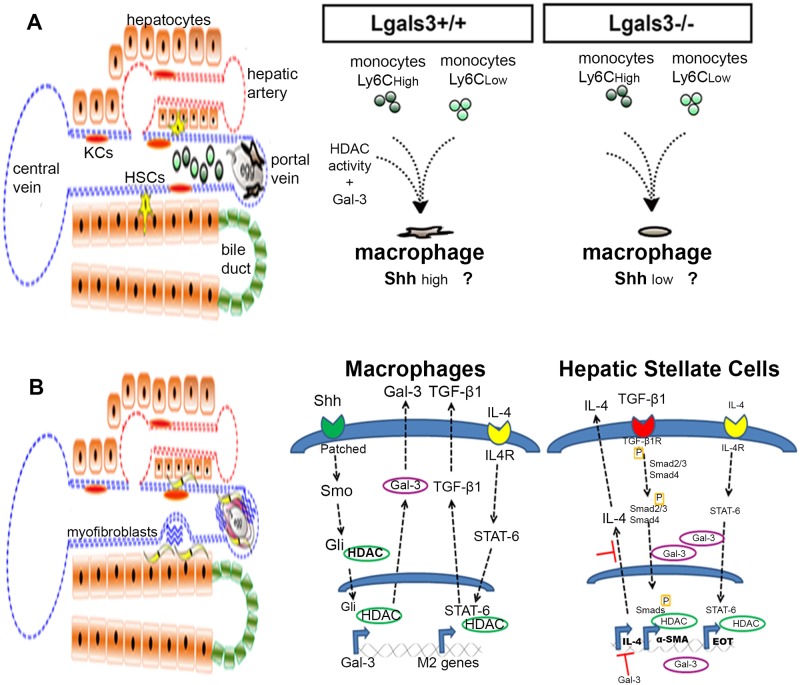
Schematic illustration of different stages of schistosomiasis and opening questions in the field. (A) Schematic illustrating liver histological architecture upon schistosomiasis infection in Lgals3+/+ and Lgals3-/- mice. Schistosomules accumulate in the hepatic portal system. Monocytes are recruited by eggs and worms antigens, becoming the central players during the first steps of the establishment of the disease. Macrophages trigger bone marrow mobilization and myofibroblast activation that, in turn, bring hepatocytes into the inflammatory process. By contrast, in Schistosoma-infected Lgals3-/- mice, macrophages are not properly activated, suggesting disturbances in monocyte recruitment, Sonic hedgehog (Shh) secretion, HDAC activity, and extracellular matrix deposition by myofibroblast cells. (B) Suggestion of possible mechanisms to be investigated in the near future.

Another interesting finding of our studies concerning the composition of inflammatory cells around the egg granulomas in Lgals3-/- mice was the predominance of eosinophils in liver granulomas compared with Lgals3+/+ mice [32]. The GR-HSCs of both groups of mice expressed similar levels of IL-5, the critical cytokines for differentiation and proliferation of eosinophils [41]. In contrast, eotaxin, a main chemoatractant chemokine for eosinophils [42], was significantly downregulated in GR-HSCs in the absence of Gal-3 (Fig 3H). In this context, we can suggest that in the absence of Gal-3, the low expression of eotaxin in GR-HSCs, even without alteration in IL-5 expression, favored the local proliferation of myeloid cells instead of the mobilization from bone marrow (Fig 4).

## Hh signaling regulates liver fibrosis during schistosomiasis

Recently, the Hh signaling pathway has been extensively investigated as a possible mediator of chronic liver inflammation and fibrosis. The Hh pathway normally plays a critical role in embryonic development and organogenesis, but in the adult, it has been shown to promote cancer development and progression [43,44,45]. In the normal adult liver, Hh signaling is switched off but could be reactivated during liver injury in HSCs, hepatocytes, and macrophages and, if activity is unchecked, may lead to liver fibrosis and cirrhosis [46,47,48]. Hh ligands can also stimulate liver progenitor cells to proliferate and secrete profibrogenic factors such as osteopontin (a matrix molecule and cytokine) and chemokines such as CXCL16 or MCP-1 that chemoattract immune cells that amplify the fibrogenic response. Conversely, inhibiting the Hh pathway significantly attenuates fibrotic outcomes [49,50], confirming the Hh pathway as a target to control liver fibrosis.

Patients with Schistosomiasis-associated portal fibrosis and portal hypertension express high levels of Hh activity in situ [47]. Specifically, the degree of Hh pathway activation correlated with fibrosis stage and was associated with macrophage phenotype [48]. Patients with schistosomiasis expressed more Hh ligands (sonic Hh [Shh] and Indian Hh [Ihh]) and Hh-target genes (Patched and Gli2) than healthy individuals and accumulated more Hh-responsive myofibroblasts and M2 macrophages, which themselves co-expressed Hh ligands, sonic Hh, and Indian Hh. These data show that soluble egg antigens (SEA) can directly stimulate liver macrophages to produce Hh ligands, which promotes alternative activation of macrophages, fibrogenesis, and vascular remodeling in schistosomiasis.

Despite similarities between the roles of Gal-3 and Hh signaling during schistosomiasis-associated liver fibrosis, it is unclear if (and how) Hh and Gal-3 interact. Interestingly, it has been demonstrated that Sufu (Suppressor of fused), a negative regulator of the Hh signal transduction pathway, interacts with Gal-3 when this lectin is translocated to the cytoplasm [51]. Both Gal-3 and Hh also exert mitogenic and fibrogenic effects during tumor progression [52]. Future studies, however, will be needed to better understand this crosstalk between HSCs and immune cells.

## The epigenetic landscape in *S*. *mansoni*

The epigenetic machinery comprises a series of protein complexes harboring catalytic activities that ultimately drive conformational changes in DNA. These changes occur through post-translational modification of histone proteins, such as histone acetylation. The level of acetylation is tightly controlled by the interplay between two histone-modifying enzymes, the histone acetyl transferases (HATs) and HDACs. The former are associated with silencing complexes, which lead to transcriptional repression, and are grouped into three different classes depending on the subcellular localization and cofactor binding [53].

HDACs control fungal activity, growth, stress responses, and virulence. For these reasons, HDACs have also been proposed as therapeutic targets in the treatment of parasitic diseases elicited by *Toxoplasma gondii*, *Plasmodium falciprum*, *Trypanosoma brucei*, and *S*. *mansoni* [54,55,56,57,58]. On the other hand, HDAC inhibitors (HDACi) have been tested in clinical trials as potential antitumor agents, which induce cell cycle arrest and tumor apoptosis [59,60]. Recently, HDACi (such as vorinostat) increase the susceptibility of uninfected CD4^+^ T lymphocytes to infection with HIV [61] and have been considered novel alternative antifungal agents [62].

During its life cycle, *S*. *mansoni* undergoes drastic changes in phenotype that are associated with epigenetic modifications, including the effects of hSirt2 inhibitors, recently considered a new antiparasite target [63]. On this basis, a Schistosome Epigenetics Consortium (targets, regulation, and new drugs) was launched to study the potential utility of HDACs in the treatment of adult schistosomiasis and to develop alternate treatments for prazinquantel-resistant Schistosoma. Indeed, findings from several studies support the role for HDAC in the Schistosoma life cycle. Class I HDACs (1, 3, and 8), for example, are overexpressed in all life cycle stages of the parasite and present a highly conserved catalytic domain, which resembles human HDACs [64].

The treatment of the schistosomula and the adult form of the parasite with HDACi in vitro also increased expression levels of caspases 3/7 (which are effector caspases) and directly correlated with the degree of acetylation on the histone H4 [57]. HDACi also induced apoptosis of the larvae and adult form via inhibition of the NFκB pathway [56]. These findings indicate that HDACi may represent a useful alternative modality in the treatment of schistosomula and/or the adult form.

It has been shown that treatment with HDACi induces an anti-inflammatory cytokine profile and a reduction in the number of classically activated macrophages (i.e., M1 markers) [65]. As such, it can be envisaged that HDACi may promote a more anti-inflammatory response by decreasing the host’s immune response to egg antigens. In turn, this dampens the inflammatory reaction, reduces hepatic injury, and abrogates liver fibrogenesis.

HDACs play an important role in macrophage differentiation and polarization [66,67]. During schistosomiasis, chromatin remodeling can be associated with macrophage activation and response following egg deposition [68,69]. Hence, it is plausible that HDACs (and HDACi) can be used to shape monocyte–macrophage phenotypes and to indirectly modify fibrosis outcomes following Schistosoma infection. Despite enthusiasm for the potential use of HDACi, it is important to consider potential off-target effects in vivo. For instance, HDACs play a critical role in many other physiological systems, and the potential loss of one function may lead to long-lasting chronic inflammatory complications.

## Possible crosstalk between Gal-3, HDACs, and Hh signaling during progressive liver fibrosis in *S*. *mansoni*-infected mice

The aggregate evidence presented suggests that Gal-3, Hh signaling, and epigenetic factors are key modulators of Schistosomiasis-associated liver fibrosis. Future research will be necessary to better understand how these factors (alone or in combination) drive liver fibrosis progression and to identify how we can target them effectively to enhance fibrosis resolution. The potential impact of these research areas is extremely relevant to public health in developing countries. Millions of dollars are spent annually to treat patients with end-stage liver and kidney diseases from *S*. *mansoni*, and current therapies are ineffective in reversing liver fibrosis. Currently, the use of praziquantel shows significant amelioration of hepatic fibrosis and offers the potential of a new chemotherapy for hepatic fibrosis resulting from schistosomiasis [70]. However, molecular mechanisms involved in this treatment are poorly understood. As early fibrosis is potentially reversible, we have highlighted promising targets and discussed strategies needed to translate these to the bedside.

We summarized our opinion in Fig 4. Liver histology of Lgals3+/+ and Lgals3-/- infected mice reveal that *S*. *mansoni* eggs are frequently trapped in the hepatic portal system, where initial signaling to monocyte recruitment plays a central role in establishing the disease. We suggested that macrophages are distinctly generated according to monocyte phenotypes mobilized from the bone marrow. During the pregranulomatous phase, monocyte recruitment is characterized by mixed Ly6C^high^ and Ly6C^low^ cells (Fig 4A). It is plausible to suggest that this mobilization is imbalanced in the absence of Gal-3, and thus, macrophage differentiation and myofibroblast activation would be significantly disturbed in the liver of Schistosoma-infected mice. Consequently, macrophages are not properly activated, suggesting that Shh secretion by myofibroblast cells would be downregulated.

The chronic phase of schistosomiasis is hallmarked by liver fibrosis, and in the absence of Gal-3, diffuse collagen fibers could be result of abnormal events on macrophages and HSCs. Here, we proposed that Shh-activated macrophages in Lgals3+/+ mice express high levels of Gal-3. It is possible that HDAC induces Gli translocation after deacetylation, as observed in tumors [71], and upregulates Gal-3 gene expression (Fig 4B). At least in part, Gal-3 is secreted to extracellular spaces and acts on distinct cell types. These cells are also responsible for M2–cytokine secretion after IL-4 stimulation [72]. We suggested that both mechanisms could be attributed to HDAC activity. Recent work from our group has shown an anti-inflammatory role for HDAC activity inhibitors, leading preferentially to an M2 macrophage phenotype [67].

Our data showed that activated HSCs obtained from the liver of Lgals3-/- mice showed reduced mRNA levels of α-SMA and eotaxin, whereas mRNA levels of IL-4 were increased in comparison to Lgals3+/+ mice. Then, we hypothesized that the TGF-β1 pathway constitutes an interesting target for future studies linking Gal-3 and HDACs regulating α-SMA synthesis (Fig 4B). Moreover, we suggested that Gal-3 and HDACs inhibit eotaxin synthesis dependent on IL-4 activation (Fig 4B). Finally, it is possible that IL-4 gene expression is regulated by mechanisms involving Gal-3, considering that data obtained from Lgals3-/- infected mice.

For this reason, it is an opening question regarding the use of these classes of inhibitors as a therapeutic approach. Indeed, considering that the first step of the infection is under the control of a macrophage, the outcome of the disease needs to be further investigated, including in the Lgals3-/- infected mice. The downmodulation of macrophages in Lgals3-/- infected mice during the first step of the infection could be directly associated with a type of fibrogranulomatous reaction in the liver and, consequently, drive profibrotic events during schistosomiasis.

## Perspectives and conclusions

Epigenetic modifiers may be useful in promoting an anti-inflammatory response. Both Gal-3 and the Hh pathway are promising antifibrotic targets. Here, we proposed that cell signaling pathways involving Gal-3, HDCAs, and Hh mechanisms could be involved with pathogenesis of schistosomiasis and consequently liver fibrosis. We suggest that these molecular mechanisms should be studied before the establishment of egg deposition, macrophage differentiation in the liver, and collagen deposition, hallmarks of the hepatic failures due to liver fibrosis in schistosomiasis.

## Methods

### Schistosomal infection

Inbred C57/bl6 (Lgals3+/+) and Lgals-3-/- mice were obtained from the colony at the Federal University of Rio de Janeiro (Brazil). Mice 4 weeks of age were infected by transcutaneous penetration of 40 *S*. *mansoni cercariae* (BH strain, Oswaldo Cruz Institute, Rio de Janeiro, Brazil). Mice were killed on day 90 post-infection, corresponding to the chronic phase.

### Ethics statement

All procedures with mice were performed in accordance with institutional guidelines (protocol number DAHEICB 070, Institute of Biomedical Sciences, Federal University of Rio de Janeiro).

### Histological preparations

For histological analyses, five animals per group were killed under anesthesia given IP (ketamine, 35 mg/kg; xylazine, 9 mg/kg, Sigma Chemical Co., St. Louis, Missouri, United States of America). The liver was removed, cut into 0.5-mm-thick slices, washed in cold saline, and fixed in Bouin’s fixative. After 6 hours of fixation, specimens were dehydrated in alcohol and embedded in paraffin. Sections of 5 μm were obtained and stained with hematoxylin and eosin, Gomori trichrome, and reticulin staining. Bright-field pictures were acquired using an Evolution MP 5.0 RTV Color camera (Media Cybernetics, Canada).

### Isolation and purification of primary murine HSCs derived from schistosome-infected mice

Mice were killed 90 days after infection. Periovular granulomas were isolated from homogenized liver tissue by sedimentation and digested by collagenase 1A (Sigma-Aldrich, Inc., St. Louis, MO, USA). The harvested cells were seeded into 25cm^2^ tissue culture flask in Dulbecco's Modified Eagle's Medium (DMEM) with 10% fetal bovine serum. Cells were subcultured by trypsinization that eliminated the trypsin-resistant granuloma macrophages. After the third passage, homogeneous HSCs derived from hepatic granulomas were obtained. These cells, named GR-HSCs, were fully described and characterized in previous studies [73].

### mRNA extraction and real-time RT-PCR

mRNA was extracted from 0.5 to 1 x 10^6^ cells using TRIzol Reagent following standard protocols. Total RNA was dissolved in water, quantified and stored at -20°C. cDNA was synthesized from 1 μg total RNA. RNA was primed with 0.5 μg oligo-(dT) primer, denatured (10 min at 64°C), and cooled on ice. The following reagents were mixed and added: 10 mM of each deoxynucleoside triphosphate (dNTP), 6 μL 5 x reverse transcriptase (RT) buffer, 0.1 mM DTT, and 200 U of MLV-RT. Reaction was performed by incubation for one hour at 37°C. During amplification, we used predesigned TaqMan Gene Expression Assays (Applied Biosystems) according to the manufacturer’s instructions for IL-5 (NCBI RefSeq: NM_010558.1), eotaxin (NCBI RefSeq: NM_011330.3), IL-4 (NCBI RefSeq: NM_021283.2), α-SMA (NCBI RefSeq: NM_007392.3), and HPRT (NCBI RefSeq: NM_013556.2). The qPCR reaction was performed with a final volume of 25 μL, containing 2 μL of cDNA, 12.5 μL of master mix (Applied Biosystems), and 250 nmol of primers and probe in optical 96-well plates. The fluorescence emission from each reaction was measured three times during the annealing/extension, and amplification plots were analyzed using software from Applied Biosystems. The gene expression data were normalized using HPRT as a housekeeping gene.

**Key learning points in the review**

- Helminthes of the genus *Schistosoma* cause a neglected tropical disease that affects approximately 240 million people, mainly in developing countries.
- *S*. *mansoni* leads to liver diseases because of egg deposition in the periportal zones, which induces a granulomatous reaction that culminates in liver fibrosis and portal hypertension.
- At the beginning of the eggs’ deposition, their antigens elicit a local production of inflammatory cytokines from resident epithelial cells and macrophages, giving rise to a macrophage-dependent granulomatous inflammatory reaction.
- The aggregate evidence presented here suggests that Gal-3, Hh signaling, and epigenetic factors are key modulators of Schistosomiasis-associated liver fibrosis modulated by macrophages in the beginning of the infection.
- Further research will be necessary to better understand how these factors, either alone or in combination, drive liver fibrosis progression and to identify how we can target them effectively to enhance fibrosis resolution. As early fibrosis is potentially reversible, we have highlighted promising targets and discussed strategies needed to translate this knowledge to the bedside.

**Top five papers**

1. Brito JM, Borojevic R (1997) Liver granulomas in schistosomiasis: mast cell-dependent induction of SCF expression in hepatic stellate cells is mediated by TNF-alpha. J Leukoc Biol 62: 389–396.
2. Henderson NC, Mackinnon AC, Farnworth SL, Poirier F, Russo FP, et al. (2006) Galectin-3 regulates myofibroblast activation and hepatic fibrosis. Proc Natl Acad Sci U S A 103: 5060–5065.
3. Oliveira FL, Frazao P, Chammas R, Hsu DK, Liu FT, et al. (2007) Kinetics of mobilization and differentiation of lymphohematopoietic cells during experimental murine schistosomiasis in galectin-3 (-/-) mice. J Leukoc Biol. 2007; 82: 300–310.
4. Pereira TA, Xie G, Choi SS, Syn WK, Voieta I, et al. (2013) Macrophage-derived Hedgehog ligands promotes fibrogenic and angiogenic responses in human schistosomiasis mansoni. Liver Int 33: 149–161.
5. Cabanel M, Brand C, Oliveira-Nunes MC, Cabral-Piccin MP, Lopes MF, et al. (2015) Epigenetic Control of Macrophage Shape Transition towards an Atypical Elongated Phenotype by Histone Deacetylase Activity. PLoS ONE 10: e0132984.
